# Relationship between crown-like structures and sex-steroid hormones in breast adipose tissue and serum among postmenopausal breast cancer patients

**DOI:** 10.1186/s13058-016-0791-4

**Published:** 2017-01-19

**Authors:** Maeve Mullooly, Hannah P. Yang, Roni T. Falk, Sarah J. Nyante, Renata Cora, Ruth M. Pfeiffer, Derek C. Radisky, Daniel W. Visscher, Lynn C. Hartmann, Jodi M. Carter, Amy C. Degnim, Frank Z. Stanczyk, Jonine D. Figueroa, Montserrat Garcia-Closas, Jolanta Lissowska, Melissa A. Troester, Stephen M. Hewitt, Louise A. Brinton, Mark E. Sherman, Gretchen L. Gierach

**Affiliations:** 1 Division of Cancer Epidemiology and Genetics, National Cancer Institute, 9609 Medical Center Drive, Bethesda, MD 20892 USA; 2 Cancer Prevention Fellowship Program, Division of Cancer Prevention, National Cancer Institute, Bethesda, MD USA; 3 Department of Radiology, School of Medicine, University of North Carolina at Chapel Hill, Chapel Hill, NC USA; 4 Independent contractor, CT(ASCP), MB(ASCP), Stamford, CT USA; 5 Mayo Clinic, Jacksonville, FL USA; 6 Mayo Clinic, Rochester, Minnesota USA; 7 Department of Obstetrics and Gynecology, Keck School of Medicine, University of Southern California, Los Angeles, CA USA; 8 Usher Institute of Population Health Sciences and Informatics, The University of Edinburgh, Medical School, Teviot Place, Edinburgh, UK; 9 Department of Cancer Epidemiology and Prevention, Cancer Center and M. Sklodowska-Curie Institute of Oncology, Warsaw, Poland; 10 Department of Epidemiology and Lineberger Comprehensive Cancer Center, University of North Carolina at Chapel Hill, Chapel Hill, NC USA; 11 Laboratory of Pathology, Center for Cancer Research, National Cancer Institute, National Institutes of Health, Bethesda, Maryland USA; 12 Breast and Gynecologic Cancer Research Group, Division of Cancer Prevention, National Cancer Institute, Bethesda, MD USA

**Keywords:** Crown-like structures, Estrogens, Hormones, Postmenopausal, Breast cancer

## Abstract

**Background:**

Postmenopausal obesity is associated with increased circulating levels of androgens and estrogens and elevated breast cancer risk. Crown-like structures (CLS; microscopic foci of dying adipocytes surrounded by macrophages) are proposed to represent sites of increased aromatization of androgens to estrogens. Accordingly, we examined relationships between CLS and sex-steroid hormones in breast adipose tissue and serum from postmenopausal breast cancer patients.

**Methods:**

Formalin-fixed paraffin embedded benign breast tissues collected for research from postmenopausal women (*n* = 83) diagnosed with invasive breast cancer in the Polish Breast Cancer Study (PBCS) were evaluated. Tissues were immunohistochemically stained for CD68 to determine the presence of CLS per unit area of adipose tissue. Relationships were assessed between CD68 density and CLS and previously reported sex-steroid hormones quantified using radioimmunoassays in serum taken at the time of diagnosis and in fresh frozen adipose tissue taken at the time of surgery. Logistic regression analysis was used to estimate odds ratios (ORs) and 95% confidence intervals (CIs) for the relationships between hormones (in tertiles) and CLS.

**Results:**

CLS were observed in 36% of benign breast tissues, with a higher frequency among obese versus lean women (54% versus 17%, *p* = 0.03). Detection of CLS was not related to individual hormone levels or breast tumor pathology characteristics. However, detection of CLS was associated with hormone ratios. Compared with women in the highest tertile of estrone:androstenedione ratio in fat, those in the lowest tertile were less likely to have CLS (OR 0.12, 95% CI 0.03–0.59). A similar pattern was observed with estradiol:testosterone ratio in serum and CLS (lowest versus highest tertile, OR 0.18, 95% CI 0.04–0.72).

**Conclusions:**

CLS were more frequently identified in the breast fat of obese women and were associated with increased ratios of select estrogens:androgens in the blood and tissues, but not with individual hormones. Additional studies on CLS, tissue and blood hormone levels, and breast cancer risk are needed to understand and confirm these findings.

**Electronic supplementary material:**

The online version of this article (doi:10.1186/s13058-016-0791-4) contains supplementary material, which is available to authorized users.

## Background

Obesity is linked to a 25% increase in the risk of developing breast cancer, and a recent meta-analysis of 82 studies found a 34% higher overall mortality risk among obese as compared with lean postmenopausal breast cancer patients [1–4]. Among postmenopausal women, most endogenous estrogen is derived from aromatization of androgens in peripheral adipose tissues, and those in the highest quintile of circulating estrogen levels experience a twofold increased risk of developing breast cancer compared with those who have the lowest levels [5, 6]. Limited data suggest that androgen levels are higher in the breast tissue than in circulation, and that concentrations are higher in benign versus malignant tissues, suggesting that local aromatization of androgens to estrogens within the breast could contribute to carcinogenesis [7].

Prior analysis of sex-steroid hormones in serum and breast adipose tissue from postmenopausal women with breast cancer including estrogen receptor (ER)-positive and ER-negative breast cancer in the Polish Breast Cancer Study (PBCS) demonstrated positive Pearson’s correlation coefficients for androstenedione (ER-positive = 0.81, ER-negative = 0.66), testosterone (ER-positive = 0.57, ER-negative = 0.44), estrone (ER-positive = 0.74, ER-negative = 0.75), and estradiol (ER-positive = 0.67, ER-negative = 0.35) levels in breast adipose tissue and in blood, suggesting that the circulating levels may provide a set-point for tissue levels [8, 9]. Thus, factors other than serum levels may account for the remaining 33–50% of the variance in adipose tissue hormone concentrations. Further, within breast adipose tissue, the ratio of testosterone to estradiol was statistically significantly lower than in serum, suggesting that hormone metabolism or other tissue factors might affect hormone concentrations locally in the breast, and could affect the behavior of immediately adjacent epithelium. Specifically, these findings support the hypothesis that aromatization of androgens to estrogens in breast fat might increase estrogen levels, and potentially exert local influences on carcinogenesis [8, 9]. We hypothesize that crown-like structures (CLS) may represent tissue factors that influence local hormone aromatization.

**Fig. 1:**
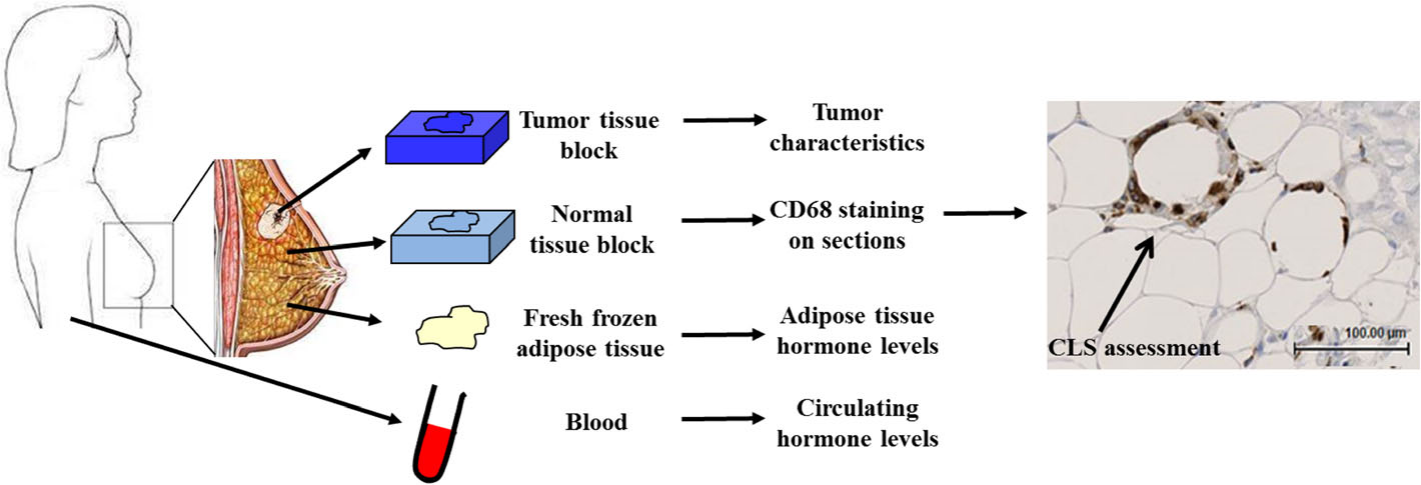
Overview of samples collected at the time of surgery and including the normal breast tissue blocks used in this analysis for crown-like structure (*CLS*) assessment. The *arrow* in the CD68 stained breast adipose tissue section shows an adipocyte engulfed by brown CD68-positive macrophages that forms the CLS

CLS are histological features in the breast adipose tissue that are recognized by the organized accumulation of CD68-positive macrophages that surround dead or dying adipocytes (shown in Fig. 1), and they may be markers of chronic inflammation. Studies using animal models and breast tissues of women suggest that their presence is associated with obesity and a pro-inflammatory, pro-carcinogenic process characterized by increased aromatase expression and activity and elevated signaling through ER-mediated pathways [10–12]. Data also suggest that the presence of CLS adversely affects outcomes among women with ER-positive breast cancers, and, in mice, CLS-related pro-inflammatory and carcinogenic processes may be reversible with weight-loss or chemopreventive agents [13, 14]. Accordingly, CLS in breast adipose tissue may represent one mechanism of increased local estrogen production, which would support the hypothesized role of local hormone levels in breast cancer development and progression.

In order to investigate whether the identification of CLS is related to concentrations and ratios of sex-steroid hormones in breast adipose tissue relative to systemic circulation, we analyzed the presence of CLS in benign breast tissue collected from surgical pathology specimens of postmenopausal breast cancer patients and compared results with previously measured hormone levels in breast fat and serum [9]. We hypothesized that tissues with the presence of CLS would be associated with elevated ratios of estrogens to androgens, consistent with elevated aromatase activity.

## Methods

### Study design and subject selection

The PBCS is a population-based, case-control study that recruited women with histopathologically confirmed in-situ and invasive breast cancer and age- and location-matched controls at five institutions in Warsaw or Łódź, Poland, between 2000 and 2003 [15]. Consenting women completed a detailed risk factor questionnaire, donated blood, and provided access to tissues for research. Briefly, this current case-only study includes a subset of 83 postmenopausal women not taking hormone replacement who underwent surgery for ER-positive/progesterone receptor (PR)-positive or ER-negative/PR-negative invasive breast cancer and for whom sex-steroid hormone measurements had been previously measured in breast adipose tissue and serum using validated methods, as described elsewhere [8, 9, 15]. These cases had not received neoadjuvant treatment and had formalin-fixed paraffin embedded (FFPE) tissues of benign breast tissue collected per protocol at the time of breast cancer surgery [9]. Benign breast tissue collected at varying distances from the tumor, and thus physically separated from the tumor, was used for CLS assessment (as outlined in Fig. 1). Tissue hormone levels were measured in fresh grossly homogeneous-appearing breast adipose tissue that was frozen in liquid nitrogen according to protocol until testing (as further described below). Thus, frozen fat used for hormone measurements was consumed by the assay and CLS were enumerated in different pieces of tissue from the same breast.

Covariate data included in this analysis were derived from the study interview as previously described [15]. Confounders evaluated included age at study enrollment (40–49 years, 50–59 years, 60–69 years, and ≥70 years), body mass index (BMI; lean <25, overweight 25–29.9, or obese ≥30 kg/m^2^), age at menarche (<13 years or ≥13 years), age at menopause (<45 years, 45–49 years, 50–54 years, or ≥55 years), age at first birth (nulliparous, <30 years, or ≥30 years), family history of breast cancer (no or yes), and previous benign breast disease (no or yes). Information on disease characteristics including tumor size (≤2 cm or >2 cm), nodal status (lymph node negative, 1–3 positive lymph nodes, or ≥4 positive lymph nodes), tumor grade (well, moderately, and poorly differentiated), and ER and PR status (positive or negative) was obtained from medical records and surgical pathology forms as previously described [15]. All participants included in this study provided consent under a protocol approved by the US National Cancer Institute and local (Polish) Institutional Review Boards.

### Analysis of crown-like structures (CLS)

To evaluate the presence of CLS, sections of designated benign tissues which were collected at varying distances from the tumor at the time of surgery and prepared as FFPE tissue blocks were sectioned and stained for CD68 (1:100; M0876, DAKO Cytomation, Carpenteria, CA, USA) using immunohistochemistry (IHC). Previously, CLS were pathologically identified using visual assessment of hematoxylin and eosin (H&E) stained tissue sections. However, more recent analysis showed that staining with the established macrophage marker CD68 increased sensitivity of CLS detection compared with H&E staining [10]; therefore, CD68 was used as a marker for the identification of CLS in this study. Briefly, following deparaffinization of FFPE tissue slides with three changes of xylene and rehydration in alcohol (100%, 95%, and 70% EtOH), slides were rinsed and underwent HIER (heat-inactivated epitope retrieval). For the HIER step, slides were placed in preheated citrate buffer (10 nmol/L; pH 6.0) for 30 min, followed by cooling for 5 min and rinsing for an additional 5 min in distilled water. Slides were then stained using the DAKO Autostainer at room temperature which included a 5-min incubation with 3% H_2_O_2_ in ethanol followed by incubation with CD68 for 30 min. Next, secondary antibody incubation was carried out using the mEnVision+/HRP-labeled polymer (K4002, DAKO Cytomation) for 15 min, and the slides were then rinsed and placed in 3,3′-diaminobenzidine (DAB+) (K3467, DAKO Cytomation) for 5 min prior to counterstaining with Modified Schmidts’s Hematoxylin for 5 min. The slides were then rinsed with water, rehydrated with alcohol, cleared in xylene, and mounted for CD68 assessment.

CD68-stained sections were scanned to generate digital images that were uploaded into SlidePath for web-based assessment (SlidePath/Leica, Dublin, Ireland). Using the lasso tool (ImageScope, Aperio), areas of adipose tissue visible on the slide were annotated. Fat area was defined as any area on the slide ≥75% adipose. Areas affected by a previous biopsy were avoided. All annotated areas were summed to determine the total fat area (mm^2^) on each slide. CD68 and CLS were visually assessed by one of the investigators (RC) in this study. Within the fat area defined by the lasso tool the number of cells that showed CD68-positive expression was counted. The number of CD68-positive cells (ImageScope, Aperio) was normalized to total fat area (mm^2^) in the same section. The presence and number of CLS were also assessed within the observed fat area on the whole tissue slide and defined as the complete encirclement of adipocytes by CD68-positive macrophages (Fig. 1). CLS presence was defined as any CLS observed on the tissue section examined. To ensure reproducibility, assessment of CLS for the study participants (*n* = 83) was compared between two independent experts (RC and MES), with a Kappa score of 0.72 for determination of CLS presence.

### Sex-steroid hormone measurement

Methods for measuring hormones in adipose tissue and serum with validation data are described in detail elsewhere [8, 9]. Briefly, adipose tissue was enzymatically digested with collagenase and then centrifuged to yield oil for hormone measurements. Partition chromatography using Celite columns was performed to elute sex-steroid hormones from serum and oil. Androstenedione, testosterone, estrone, and estradiol were quantified by radioimmunoasssy (RIA) [8].

### Statistical analysis

Frequencies and percentages were used to describe selected characteristics of the study population. Sex-steroid hormone levels from both serum and tissue were transformed to the natural logarithm scale to normalize values. Relationships between the number of CD68-positive macrophages per unit fat area and patient and clinical characteristics were analyzed using Mann Whitney *U* and Kruskal-Wallis analyses. To assess whether patient and clinical characteristics of the tumor were associated with CLS, we evaluated relationships using logistic regression to determine odds ratios (ORs) and 95% confidence intervals (CIs) for the presence of CLS. The number of CD68-positive macrophages and CLS per unit fat area were related to tissue and circulating hormones using Spearman’s correlation analysis. Covariates that had a significant association with CLS through chi-square or Kruskal-Wallis tests were adjusted for in multivariable analysis.

We examined whether tertiles of hormone levels were associated with the presence of CLS using logistic regression, with CLS as the outcome variable and tertiles of each hormone examined as explanatory variables. Models were adjusted for age and BMI, factors previously identified as being associated with CLS [16]. Models were also adjusted for fat area, and the results of both unadjusted and adjusted models are presented. Potential effect modification of the association between tissue and blood hormone levels with the presence or absence of CLS was examined by including an interaction term in linear regression models. All analyses were conducted in SAS v9.3 (2011, SAS Institute).

## Results

### Characteristics of the study population

The mean ± SD age of participants included in this analysis was 61 ± 8.40 years, the mean BMI was 29 ± 5.77 kg/m^2^, 72% of carcinomas were ER-positive/PR-positive, and the majority of women had moderately differentiated tumors (58%) at diagnosis, characteristics which were similar to those of the larger total of postmenopausal women who provided frozen tissue specimens from Warsaw [8, 9]. The distribution of breast cancer risk factors and pathological characteristics of tumor samples are shown in Additional file 1 (Table S1).

### Distribution of CD68 and CLS in postmenopausal breast adipose tissue

CD68-positive cells were detected in all breast cancer cases examined in this study (median number of CD68-positive cells = 150, range: 5–1237) (Table 1). CLS (Fig. 1) were identified in 36% (*n* = 30/83) of the cases examined.

**Table 1:** Distribution of CD68-positive macrophage cells and CLS in postmenopausal women diagnosed with invasive breast cancer (*n* = 83)

	*n* (%)	No. of CD68-positive cells	*p* value	No. of CD68-positive cells per 100 mm^2^ fat area	*p* value
Overall	83 (100)	150.0 (5–1237)		13 (1–737)	
CLS-positive	30 (36)	250.5 (34–1237)	<0.001	20 (2–406)	0.23
CLS-negative	53 (64)	113.0 (5–522)		10 (1–737)	
ER-positive					
CLS-positive	21 (35)	253.0 (34–1237)	<0.001	19 (3–140)	0.69
CLS-negative	39 (65)	109.0 (5–481)		12 (2–364)	
ER-negative					
CLS-positive	9 (39)	188.0 (106–588)	0.06	39 (2–406)	0.16
CLS-negative	14 (61)	123.5 (6–522)		10 (1–737)	

Values are shown as median (range) unless otherwise indicated

*p* values (Mann Whitney *U* test) are shown for comparison of CLS-positive versus CLS-negative

*CLS* crown-like structures, *ER* estrogen receptor

### Relationship of breast cancer risk factors and tumor characteristics with number of CD68-positive cells and CLS among postmenopausal breast cancer cases

In univariate analyses, the number of CD68-positive macrophages per unit area of fat was not significantly related to patient and clinical characteristics or to hormone levels in serum or adipose tissue (Additional file 1: Table S1). The number of CLS per unit area of fat was significantly positively correlated with BMI and age at menopause, and inversely correlated with age at first birth (*p* < 0.05 for each correlation). CLS number per unit area of fat was also inversely correlated with levels of androstenedione both in tissue (*p* < 0.02) and in blood *p* = 0.06). Furthermore, the number of CLS per unit area of fat was significantly positively correlated with the ratio of estrone to androstenedione both in tissue and in blood (*p* < 0.05 for each correlation; Additional file 1: Table S1).

In analyses adjusted for age and fat tissue area, the frequency of CLS-positive cases was significantly higher among obese than normal weight women (unadjusted OR 5.61, 95% CI 1.46–21.53; adjusted OR 4.63, 95% CI 1.08–19.83; Table 2). Analyses were adjusted for age as CLS were previously found to be associated with menopausal status [16]. Associations with other patient and tumor characteristics were not statistically significant.

**Table 2:** Relation of clinicopathological characteristics with presence of CLS among postmenopausal women with invasive breast cancer (*n* = 83)

	CLS-positive *n* (%)	CLS-negative *n* (%)		Unadjusted		Adjusted^b^	
Patient characteristic			*p* value^a^	OR	95% CI	OR	95% CI
Age			0.30				
40–49 years	1 (3.3)	7 (13.0)		0.34	0.04–3.20	0.31	0.03–3.12
50–59 years	8 (26.7)	19 (35.2)		Reference		Reference	
60–69 years	12 (40.0)	18 (33.3)		1.68	0.55–5.08	1.17	0.35–3.91
≥70 years	9 (30.0)	10 (18.5)		2.14	0.63–7.26	1.63	0.45–6.00
BMI category			**0.03**				
Lean	4 (14)	19 (36)		Reference		Reference	
Overweight	13 (43)	23 (43)		2.69	0.75–9.61	1.93	0.50–7.40
Obese	13 (43)	11 (21)		**5.61**	**1.46**–**21.53**	**4.63**	**1.08**–**19.83**
Age at menarche			0.28				
<13 years	9 (31)	10 (19)		Reference		Reference	
≥13 years	20 (69)	43 (81)		0.52	0.18–1.47	0.40	0.12–1.32
Age at menopause			0.09				
<45 years	2 (7)	6 (11)		Reference		Reference	
45–49 years	8 (27)	25 (47)		0.96	0.16–5.74	1.50	0.21–10.44
50–54 years	13 (43)	18 (34)		2.17	0.38–12.50	2.95	0.42–20.68
≥55 years	7 (23)	4 (8)		5.25	0.70–39.48	6.19	0.63–60.73
Age at first birth			1.00				
Nulliparous	5 (17)	10 (19)		Reference		Reference	
<30 years	20 (66)	35 (66)		1.14	0.34–3.82	1.08	0.28–4.17
≥30 years	5 (17)	8 (15)		1.25	0.27–5.89	1.42	0.26–7.93
Family history of breast cancer			0.53				
No	24 (80)	46 (87)		Reference		Reference	
Yes	6 (20)	7 (13)		1.64	0.50–5.44	2.91	0.71–11.96
Benign breast disease			0.74				
No	25 (89)	44 (85)		Reference		Reference	
Yes	3 (11)	8 (15)		0.66	0.16–2.72	1.09	0.22–5.35
Tumor size			1.00				
≤2 cm	15 (52)	26 (49)		Reference		Reference	
>2 cm	14 (48)	27 (51)		0.90	0.36–2.22	0.62	0.22–1.75
Nodal status			0.22				
No	20 (67)	26 (49)		Reference		Reference	
1–3	7 (23)	15 (28)		0.61	0.21–1.77	0.38	0.11–1.41
≥4	3 (10)	12 (23)		0.33	0.08–1.31	0.44	0.10–2.00
ER status			0.80				
Positive	21 (70)	39 (74)		Reference		Reference	
Negative	9 (30)	14 (26)		1.19	0.44–3.22	1.09	0.35–3.39
PR status			0.80				
Positive	21 (70)	39 (74)		Reference		Reference	
Negative	9 (30)	14 (26)		1.19	0.44–3.22	1.09	0.35–3.39
Diagnosis			0.65				
In situ and invasive	17 (57)	33 (62)		Reference		Reference	
Invasive only	13 (43)	20 (38)		1.26	0.51–3.14	1.22	0.45–3.29
Grade			0.70				
Well differentiated	4 (13)	11 (21)		Reference		Reference	
Moderately differentiated	19 (64)	29 (55)		1.80	0.50–6.49	1.32	0.33–5.24
Poorly differentiated	7 (23)	13 (24)		1.48	0.34–6.42	1.68	0.33–8.55

^a^Fisher's exact test. Significant values are shown in bold

^b^Adjusted for age and BMI (age was adjusted for BMI only, BMI was adjusted for age only) and fat area

*BMI* body mass index, *CI* confidence interval, *CLS* crown-like structures, *ER* estrogen receptor, *OR* odds ratio, *PR* progesterone receptor

### Relationship of CLS presence with sex-steroid hormone levels in adipose tissue and serum of postmenopausal breast cancer cases

Most relationships of individual hormone levels in adipose tissue and blood with the presence of CLS were not significant, with a few exceptions (Table 3). In adjusted analyses, estrone levels in adipose tissue were positively associated with the presence of CLS, but only in the middle as compared with the highest estrone tertile (adjusted OR_T2vsT3_ 4.35, 95% CI 1.06–17.86).

**Table 3:** Associations between tissue and blood levels of sex-steroid hormones measured, and presence of CLS among postmenopausal women with invasive breast cancer (*n* = 83)

	Tissue (pg/g)						Blood (pg/ml)					
			Unadjusted		Adjusted^a^				Unadjusted		Adjusted^a^	
	CLS-positive	CLS-negative	OR	95% CI	OR	95% CI	CLS-positive	CLS-negative	OR	95% CI	OR	95% CI
*Individual hormones*												
Androstenedione												
Tertile 3	7	21	Reference		Reference		7	21	Reference		Reference	
Tertile 2	10	17	1.77	0.84–8.07	1.67	0.47–5.93	11	16	2.06	0.65–6.51	0.46	0.13–1.62
Tertile 1	13	15	2.60	0.84–8.07	3.13	0.88–11.18	12	16	2.25	0.72–7.01	0.95	0.28–3.20
*p* trend			0.28		0.08				0.17		0.23	
Testosterone												
Tertile 3	9	19	Reference		Reference		7	17	Reference		Reference	
Tertile 2	13	14	1.96	0.66–5.86	2.29	0.66–7.91	10	16	1.79	0.56–5.74	1.86	0.50–7.00
Tertile 1	8	20	0.85	0.27–2.64	1.20	0.30–4.80	13	19	2.32	0.75–7.19	3.33	0.91–12.20
*p* trend			0.78		0.70				0.15		0.07	
Estrone												
Tertile 3	10	18	Reference		Reference		10	17	Reference		Reference	
Tertile 2	15	12	2.25	0.76–6.65	**4.35**	**1.06**–**17.86**	10	16	1.06	0.35–3.23	1.83	0.46–7.22
Tertile 1	5	23	0.39	0.11–1.35	1.01	0.20–5.10	9	19	0.81	0.27–2.45	2.16	0.53–8.79
*p* trend			0.17		0.90				0.70		0.29	
Estradiol												
Tertiles 3	12	15	Reference		Reference		10	17	Reference		Reference	
Tertiles 2	9	18	0.63	0.21–1.88	0.69	0.20–2.39	8	14	0.97	0.30–3.12	1.41	0.38–5.30
Tertiles 1	8	20	0.50	0.16–1.53	0.61	0.17–2.20	12	21	0.97	0.34–2.79	1.49	0.44–5.08
*p* trend			0.22		0.46				0.96		0.53	
*Hormone ratios*												
Estrone:Androstenedione												
Tertiles 3	17	11	Reference		Reference		13	14	Reference		Reference	
Tertiles 2	9	18	**0.32**	**0.11**–**0.97**	0.30	0.08–1.17	10	17	0.63	0.21–1.88	1.00	0.29–3.47
Tertiles 1	4	24	**0.11**	**0.03**–**0.40**	**0.12**	**0.03**–**0.59**	6	21	**0.31**	**0.10**–**1.00**	0.59	0.15–2.29
*p* trend			**<0.01**		**0.01**				0.05		0.46	
Estradiol:Testosterone												
Tertiles 3	12	16	Reference		Reference		17	12	Reference		Reference	
Tertiles 2	9	18	0.67	0.22–1.99	0.46	0.13–1.64	7	18	**0.28**	**0.09**–**0.86**	0.30	0.08–1.22
Tertiles 1	8	19	0.56	0.18–1.71	0.38	0.10–1.39	6	21	**0.20**	**0.06**–**0.65**	**0.18**	**0.04**–**0.72**
*p* trend			0.31		0.15				**0.01**		**0.02**	

Significant values are shown in bold

^a^Adjusted for age, BMI, and fat area

*BMI* body mass index, *CI* confidence interval, *CLS* crown-like structures, *OR* odds ratio

**Fig. 2:**
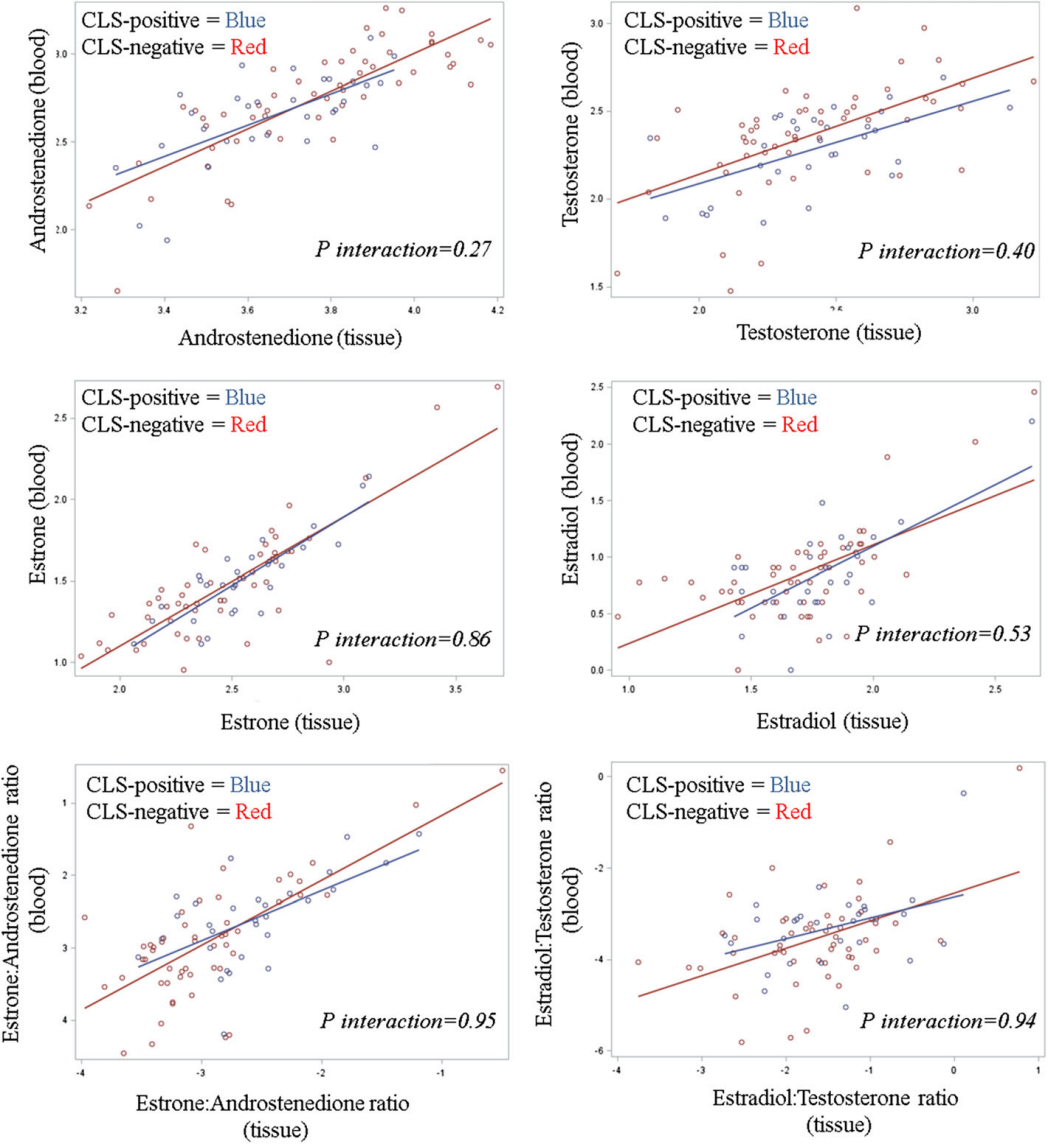
Effect of the presence of crown-like structures (*CLS*) on the correlation between sex-steroid hormone levels measured in the circulation and in breast adipose tissues

In contrast to the generally null relationships between individual hormone levels and CLS status, ratios of estrogens to precursor androgens were significantly associated with CLS status. Compared with women whose estrone:androstenedione ratio in fat was in the highest tertile, women in the lowest tertile were less likely to be CLS-positive (adjusted OR_T1vsT3_ 0.12, 95% CI 0.03–0.59; *p*_*trend*_ = 0.01). In serum, similar relationships to those observed in fat were found in univariate but not in multivariate analyses for the estrone:androstenedione ratio. In addition, in serum, as the ratio of estradiol:testosterone decreased, CLS-positivity significantly decreased as well (adjusted OR_T1vsT3_ 0.18, 95% CI 0.04–0.72; *p*_*trend*_ = 0.02). Hormone levels and ratios of hormone levels in adipose tissue and serum were positively correlated, but these relationships were not significantly modified by CLS status (Fig. 2).

## Discussion

The aromatase enzyme is present in adipose tissue throughout the body where it converts androgens to estrogens, providing the main source of postmenopausal estrogens. Similar to prior reports, we showed that CLS were more frequently found among obese compared with lean women, and therefore might have a greater effect on hormone levels among obese women, a group which is known to experience an increased risk of hormone receptor-positive breast cancer [10, 16, 17] and adverse breast cancer prognosis [17–19].

Our analysis demonstrates that the presence of CLS in breast adipose tissue of postmenopausal patients with breast cancer is associated with increased ratios of estrogens to androgens in breast fat and serum, consistent with data showing that these microscopic inflammatory lesions are related to increased aromatase activity [10–12]. However, we were unable to directly measure hormones in the same piece of tissue in which CLS was assessed. Thus, we cannot exclude the possibility that there are important changes in hormone levels in tissues immediately surrounding CLS which could alter the microenvironment at the millimeter or submillimeter scale.

In addition, the number of CD68-positive cells was higher in cases that were CLS-positive. This association was not statistically significant after adjusting for fat tissue area; therefore, our data do not support evaluating CD68-positive cells as a surrogate of CLS. In addition, little is known about the role of CD68 in the functioning of CLS beyond forming the organized CLS. Although CD68 counts are potentially evaluable by image analysis of stained sections, recognition of CLS was carried out using subjective visual assessment [20]. Among postmenopausal women, the ovaries and adrenals continue to synthesize androgens which can be converted to biologically active estrogens in peripheral tissues. The regulation of enzymes involved in this process is complex and not well understood. Within breast tissues, multiple mechanisms may affect hormone levels, including uptake from the circulation, inter-conversion of different hormones (e.g., between estrone and estradiol), and synthesis in epithelium, stroma, or adipose tissue, the latter being a key site of aromatization of androgens to estrogens [21]. The percentage of the breast comprised of adipose tissue increases with age, and increased adipose tissue has been associated with increased breast epithelial area [22, 23]. The presence of CLS was significantly associated with increased ratios of estrone to androstenedione in breast adipose tissue independent of age, BMI, and fat area on the slide. Our association between the presence of CLS and higher levels of estrogens relative to androgens in serum and breast adipose suggests that uptake from the circulation and the local conversion of androgens to estrogens both influence the local hormonal milieu enveloping at-risk epithelium and cancer. However, CLS status was generally not associated with absolute concentrations of individual hormone levels measured in tissue or blood, although these analyses were limited by small numbers of CLS-positive cases.

Accordingly, it remains uncertain whether the presence of CLS in breast fat implies information about risk for hormone receptor-positive breast cancer beyond that provided by measuring hormone levels in serum. In line with this, similar proportions of ER-positive (35%) and ER-negative (39%) breast cancer patients were positive for the presence of CLS. Furthermore, our findings of no association between CLS and ER status are in agreement with Iyengar and colleagues [17], and suggest that CLS may also be influencing additional inflammatory mechanisms associated with increased BMI. However, even if detection of CLS does not inform the magnitude of breast cancer risk, it may have implications for understanding mechanisms of hormonal carcinogenesis.

Our results showing no association between the presence of CLS and breast tumor clinical characteristics are largely consistent with prior studies. A recent analysis by Iyengar and colleagues that included a retrospective cohort of women who underwent mastectomy following a breast cancer diagnosis also found no relationship between the presence of CLS and tumor size, grade, and histology [17]. However, in that cohort, the presence of CLS was significantly associated with worse distant relapse-free survival over a median follow-up of 23 months [17]; the association remained significant after adjusting for potential confounders including BMI. These findings may provide insight into why obese postmenopausal women suffer poorer breast cancer mortality compared with lean postmenopausal women [17–19]. Furthermore, although we did not examine the relationship between CLS and prognosis in this analysis due to a limited sample size, we and others have also detected CLS in non-obese women; 14% of our CLS-positive group was lean [17]. Additional studies are needed to understand the biological importance of CLS in non-obese women, in particular with respect to hormone levels and risk of hormonally driven cancers [17].

The focus of our analysis was to further understand the relationship between CLS and sex-steroid hormones. However, CLS may also have additional roles in breast cancer development and progression other than those addressed in this analysis. For example, in the same study described above, Iyengar and colleagues observed significant associations between CLS and clinical features of metabolic syndrome in addition to obesity including hyperinsulinemia, hyperglycemia, and hypertriglyceridemia [17]. These findings were observed in two independent patient populations included in their study, suggesting that CLS may also influence breast cancer outcomes through alternative mechanisms.

A key strength of our analysis was our ability to relate local hormone levels from the breast fat tissue to CLS presence, though a limitation of this approach was that CLS assessment was not carried out on the same frozen tissue piece from which hormone measurements were obtained. This is an important limitation because our analysis assumes that hormone levels within the sampled adipose tissue are representative of levels within normal tissue near to the tumor, where CLS were characterized. It is possible that other histological features characteristic of the microenvironment are influencing either the hormonal levels or CLS presence. Although technically challenging, future efforts comparing CD68 staining of frozen sections and hormonal measurements in the same tissue specimens could prove informative.

In our analysis, we did not differentiate between M1 and M2 macrophages, which could have implications for cancer risk or prognosis. M1 macrophages mediate the release of many pro-inflammatory cytokines including interleukin 1 beta and tumor necrosis factor-alpha. M2 macrophages are largely involved in immunosuppression (anti-inflammatory) [24]. Our analysis also relied on visual assessment of CLS. However, more recent applications of novel imaging technologies, including Raman spectroscopy, have successfully detected CLS in ex vivo fresh frozen non-cancerous tissue from mouse models as well as women without cancer, and may enable future objective high-throughput assessments of CLS [25]. The use of such imaging methods will have important implications for the translation of CLS assessment into a clinical setting [25].

An additional limitation of our study was the limited sample size that had fresh frozen adipose available for inclusion. Furthermore, this analysis of women with prevalent invasive carcinoma precluded the separation of etiologic influences from secondary effects related to the presence of tumor. However, the benign breast samples analyzed in this study, which were removed by visual inspection of surgical pathology specimens, were collected from areas that were physically separated from the tumors as outlined in Fig. 1. Future studies examining the presence and function of CLS in cancer-free women, especially in the setting of obesity, are needed to determine if CLS are involved in breast cancer development. An additional extension of these studies among women with cancer will also help to further determine the relationship between CLS and breast cancer prognosis. Expanding on studies investigating the prognostic and predictive importance of CLS is of interest because obesity is associated with increased mortality among breast cancer cases [17, 18].

## Conclusion

In summary, our data show that the presence of CLS in breast adipose tissue of breast cancer patients is related to increased ratios of estrogen to androgen levels in both breast adipose tissue and in the circulation. Our findings are consistent with the previously reported findings that CLS are associated with obesity and increased aromatase activity that alters hormone ratios in postmenopausal breast tissue, a frequent site of hormonal carcinogenesis. Additional research is needed to determine why some women, but not others, develop CLS and what role CLS may play in breast cancer risk among postmenopausal women.

## Additional file

Additional file 1: Table S1. Select patient and clinical characteristics of women included in this study as well as the relationship between the number of CD68-positive macrophages and number of CLS (per unit area of fat) and hormones. (DOCX 25 kb)

## Abbreviations

BMI: Body mass index
CI: Confidence interval
CLS: Crown-like structures
ER: Estrogen receptor
FFPE: Formalin-fixed paraffin embedded
H&E: Hematoxylin and eosin
IHC: Immunohistochemistry
OR: Odds ratio
PBCS: Polish Breast Cancer Study
PR: Progesterone receptor
