# Supplementary material for: Relationship between crown-like structures and sex-steroid hormones in breast adipose tissue and serum among postmenopausal breast cancer patients
Source: Breast Cancer Res. 2017 Jan 19;19:8. doi: 10.1186/s13058-016-0791-4 (PMC5244534; doi:10.1186/s13058-016-0791-4)
Supplement: Additional file 1: Table S1. — Select patient and clinical characteristics of women included in this study as well as the relationship between the number of CD68-positive macrophages and number of CLS (per unit area of fat) and hormones. (DOCX 25 kb) [file 13058_2016_791_MOESM1_ESM.docx]

Supplementary Table 1. Select patient and clinical characteristics of women included in this study as well as the relationship between the number of CD68-positive macrophages and number of CLS (per unit area of fat) and hormones.

***Continued on the next page…….***
